# Physical exercise/sports ameliorate the internet addiction from college students during the pandemic of COVID-19 in China

**DOI:** 10.3389/fpubh.2023.1310213

**Published:** 2023-12-21

**Authors:** Peiling Cai, Junren Wang, Peng Ye, Xiaoming Feng, Gaoqiang Yang, Chao Huang, Xinwei Chen, Brett D. Hambly, Shisan Bao, Shengxiang Liang

**Affiliations:** ^1^School of Preclinical Medicine, Chengdu, China; ^2^Clinical Medical College & Affiliated Hospital, Chengdu University, Chengdu, China; ^3^Health Management Center, Guilin, China; ^4^Guangxi Key Laboratory of Metabolic Reprogramming and Intelligent Medical Engineering for Chronic Diseases, The Second Affiliated Hospital, Guilin Medical University, Guilin, China

**Keywords:** internet addiction, college students, physical excise, disciplines, China

## Abstract

The rapid advancement of modern technology has significantly driven progress in various IT-related activities, resulting in a substantial increase in internet penetration rates, particularly among college students. The utilization of the internet has become one of the most essential tools in our modern society. However, internet addiction (IA) has emerged as a serious concern, particularly among college students, adversely affecting academic performance and having significant psychological and psychiatric implications. The aim of the current study was to determine the impact of physical exercise, gender and academic year on IA among college students. In the present study, we investigated internet usage, engagement in sports activities, and academic performance among college students from Western, Middle, and Eastern regions of Chinese universities. It’s noteworthy that most of the respondents were freshmen. Our findings indicate that freshmen students were more susceptible to experiencing IA. Approximately 75% of students engaged in leisure sports activities, revealing an inverse correlation between sports activity and IA. This correlation aligns with the level of sports involvement, emphasizing the potential benefits of physical activity in mitigating IA. However, our study did not uncover any correlation between geographic location and the occurrence of IA, nor did it find differences between medical and non-medical students. Furthermore, our study revealed no significant variations in IA among students from different ethnic backgrounds. The underlying mechanism of IA is being currently determined. Our data suggest that physical exercise, gender, and academic year have a significant impact on IA among college students.

## Introduction

The rapid advancement of modern technology has greatly propelled the progress of various IT-related activities in recent decades. This advancement can be attributed to the increasing affordability and accessibility of the internet, bringing substantial benefits to society, including cashless shopping, online registration, and ticketing. According to the 51st Statistical Report from the China Internet Network Information Centre (CNNIC), the number of internet users in China had reached 1.067 billion by December 2022, marking an increase of 35.49 million compared to December 2021, resulting in an internet penetration rate of 75.6% (1). Notably, a significant portion of these internet users, particularly among college students, accounted for 21.0% (1). Consequently, the utilization of the internet has emerged as one of the most essential tools in our modern society.

While the responsible use of the internet can enhance people’s lives in terms of enjoyment and reliability, some individuals fall into the trap of internet addiction (IA) (2, 3). This not only hampers academic performance but also gives rise to profound psychological and psychiatric issues among the students (2) As a result, the consequences of IA extend well beyond the surface, causing significant harm.

The simultaneous emergence of COVID-19 outbreaks, resulting from the infection of the SARS-CoV-2 virus, was initially identified in Wuhan in December 2019 (4). In an effort to contain and eradicate the transmission of the SARS-CoV-2 virus, authorities declared a state of emergency on January 25, 2020. This declaration led to the implementation of measures such as maintaining social distance, locking down non-essential facilities (4, 5), and mandating the wearing of facial masks.

As a response, Chinese authorities adopted a zero-tolerance policy, followed by a dynamic zero policy (6), in confronting one of the most perilous challenges of the millennium. Subsequently, the Minister of Education in China recommended suspending in-person classes but not suspending lessons altogether, transforming traditional face-to-face teaching into digital online instruction. This directive encouraged individuals to stay at home and engage in remote work, which, in turn, became an additional contributing factor to the rise in IA. Reports indicate that over 35% of the general population in China experienced IA during the COVID-19 lockdown [3]. Furthermore, it has been observed that complete lockdowns have had significant psychological and psychiatric impacts on the general population (7), notably increasing the risk of IA during the COVID-19 pandemic in China (3).

In light of the pandemic outbreak, universities transitioned from in-person to online teaching methods, resulting in a heightened frequency and prolonged duration of college students’ online activities. Research has demonstrated a substantial increase in anxiety, stress, and depression among both the community and healthcare professionals in Wuhan during the complete lockdown (8).

Surprisingly, our earlier research findings indicate that the academic performance of first-year medical students (9) and nursing students (10) has not only declined but also shown improvements at certain levels in online teaching during the lockdown. Admittedly we did not explore any psychological issues from these two studies.

Furthermore, the COVID-19 lockdown and the shift to online teaching prompted many students to engage in IT-related activities, potentially making them susceptible to IA or exacerbating the problem for those already addicted to IT. To address IA, it has been proposed that physical exercise can play a significant role in alleviating mild-to-moderate depression and anxiety in individuals (11). Additionally, it may also enhance mental activity to some extent. However, the connection between IA, physical exercise, and subsequent academic performance among college students, especially during the COVID-19 lockdown period, warrants further investigation.

However, there is a need for clarification regarding IA among college students in the past 1–3 years. Consequently, we conducted a recent survey to assess the preventive impact of engaging in sports exercise and the intensity of exercise on IA. The aim of the current study was to determine the impact of physical exercise, gender and academic year on IA among college students.

## Methods

### Sample size of research participants

In this study, we employed a convenience sampling technique and conducted a cross-sectional survey. The sample comprised 1,087 undergraduate students drawn from over ten universities, including but not limited to Chengdu University, Qinghai University, Chengdu Medical College, Chongqing University, Sichuan University, Sichuan Normal University, Southwest Medical University, North Sichuan Medical College, Guilin Medical University, Hubei University, Nanchang University, Tsinghua University, Zhejiang University, Xiamen University, Fudan University, and Shanghai Jiao Tong University, among others, representing diverse regions of China. We utilized PASS 2021 software for the analysis. To account for potential inefficiencies, a minimum of 1,145 students should have been included, assuming a 5% margin of error. This study has been approved by the Human Ethic Committee, The Second Affiliated Hospital, Guilin Medical University, Guilin, China.

### Data collection

This research was carried out during March and April 2023, in the midst of a significant COVID-19 pandemic outbreak in China. We distributed an online questionnaire survey to undergraduates using the Questionnaire Star platform.^1^ A total of 2,115 questionnaire sheets were distributed, and we received 2,108 valid responses, yielding an impressive effective response rate of 99.7%. Only seven questionnaires were excluded from the analysis due to incorrect or unclear answers.

### Questionnaire

The questionnaire utilized in this study was developed through a comprehensive review of relevant literature and encompassed both Independent Variables (IV) and Dependent Variables (DV). The Independent Variables (IVs) encompassed various sociodemographic characteristics of the students, including age, gender, ethnicity, discipline, grade, geographic location of the university (East, Middle, or West of China), Double 1st-Class status, as well as aspects related to physical exercise, such as participation in exercise and physical activity levels. The Dependent Variable (DV) centered on Internet Addiction.

## Scales for questionnaire

### Physical activity rating scale

The assessment of physical exercise levels was conducted using the Physical Activity Rating Scale (PARS-3), originally developed by Kimio Hashimoto, a psychologist at Kyushu University in Japan. It was subsequently adapted into a revised Chinese version by Liang (12). The PARS-3 comprises three items, which include exercise intensity, duration time, and exercise frequency during the past month. Each item is rated using a 5-point Likert scale. Specifically, the exercise intensity and exercise frequency items are assigned scores ranging from 1 to 5, while the duration time item is scored from 0 to 4.

The total physical exercise score was computed using the formula: total physical exercise score = exercise intensity × duration time × exercise frequency, resulting in a total score ranging from 0 to 100 points (13, 14). Higher scores indicate a higher level of physical exercise (15). Additionally, the physical activity level was categorized into three levels based on Liang’s (12) classification: low level (≤ 19 points), moderate level (20–42 points), and high level (≥ 43 points). The Cronbach’s α coefficient for the PARS-3 scale in this study was 0.633.

### Internet addiction scale

All participating students in the study completed the Internet Addiction Test (IAT) to gauge the severity of compulsive internet surfing. The IAT, developed by Young (16), is widely recognized in international research and has exhibited strong validity and reliability in prior investigations (3, 17). The IAT comprises 20 items, each rated on a 5-point Likert scale (1 = rarely, 2 = occasionally, 3 = frequently, 4 = often, and 5 = always). The total score spans from 20 to 100 points (18), with scores categorized into two primary groups: average users (< 40 points) and problematic users (≥ 40 points) (19). Furthermore, the severity of IA can be classified as normal (20–39 points), mild (40–59 points), moderate (60–79 points), and severe (80–100 points) (18). The scale exhibited excellent reliability in the present sample, with a Cronbach’s α coefficient of 0.928, signifying a high level of internal consistency among the items.

### Statistical analysis

Since the measurement data did not conform to a normal distribution, it was presented as the median and interquartile range (IQR) [M (P25, P75)]. To assess the relationships between the two data groups, the Mann–Whitney U test was employed. Categorical data was expressed in terms of frequency (n) and percentage (%), and differences between two or multiple groups were detected using the chi-square test or Fisher’s exact test.

To investigate the association between exercise participation and Internet Addiction (IA), we conducted Modified Poisson regression analysis (20, 21). This analysis allowed us to examine the relative risk (RR) and 95% confidence interval (CI) of the dependent variable (DV) in relation to the independent variables (IV) (22). We adjusted for age, gender, ethnicity, grade, geographic location of the university, and Double 1st-Class status as part of the analysis. All statistical analyses were carried out using IBM SPSS Statistics 26.0, and a value of p less than 0.05 (two-sided) was considered statistically significant.

## Results

### Participant characteristics

We received a total of 2,108 valid responses from college students. Table 1 offers a comprehensive summary of the demographic characteristics of these students. The ages of the college students ranged from 18 to 20 years, with a median age of 19 years. The proportion of females was 1.2 times higher than that of males. Furthermore, the percentage of Han ethnicity was significantly higher, with a 146.3-fold difference compared to the proportion of minority ethnicities. Additionally, the ratio of Non-Double 1^st^-class universities was 8.0 times higher than that of Double 1^st^-class universities.

**Table 1:** Overall descriptive statistics for variables (*n* = 2,108).

Variables		Median (*n)*	IQR (P25, P75) (%)
Age		19.0	(18.0, 20.0)
Sex	Men	957	45.4
	Women	1,151	54.6
Ethnic	Han	1973	93.6
	Minority	135	6.4
Double 1^st^ -class	Non	1875	88.9
	Yes	233	11.1
University location	East	219	10.4
	Middle	150	7.1
	West	1739	82.5
Discipline	Medicine	977	46.3
	STEM	598	28.4
	Others	533	25.3
Grade	1^st^	1,551	73.6
	2^nd^	297	14.1
	3^rd^	133	6.3
	4^th^	105	5.0
	5^th^	22	1.0
Leisure sports participation	No	353	16.7
	Yes	1755	83.3
Exercise intensity	Low (≤19)	1,346	63.9
	Moderate (20–42)	459	21.8
	High (≥43)	303	14.4

STEM: Science, Technology, Engineering and Math.

Regarding the geographical distribution of universities, the West of China had the highest representation, whereas the Middle of China had the lowest proportion. Among the various disciplines, Medicine had the highest representation, accounting for 46.3% of the participants, followed by STEM (Science, Technology, Engineering, and Math) at 28.4%, with other disciplines making up 25.3%. Grade 1 undergraduates constituted most respondents, comprising 73.6% of the total, in comparison to students from other grade levels.

Regarding other factors, a substantial proportion of students (83.3%) indicated their participation in leisure sports, a figure approximately 5 times higher than those who did not engage in such activities. Furthermore, 63.9% of students were categorized as having a low level of exercise intensity, which was roughly 2.9 times more prevalent than those classified with a moderate level and 4.4 times more prevalent than those with a high level of exercise intensity.

### Overall situation of internet addiction

The Internet Addiction Test (IAT) scores for all participants had a median value of 41.0, with a range spanning from 32.0 to 51.0. This score distribution indicated that the severity of IA was generally classified as mild. Based on the IAT scores, 55.8% of the participants were identified as suffering from IA, which was approximately 1.3 times more prevalent than the proportion of Non-Internet Addiction (NIA) cases. Among the IA participants, 44.4% were categorized as having a mild level of addiction, a significantly higher percentage than those classified with moderate or severe addiction levels (Table 2).

**Table 2:** Internet addiction among college students (*n* = 2,108).

Types		Median / n	IQR (P25, P75)/(%)
IAT Scores		41.0	(32.0, 51.0)
NIA (<40)		931	44.2
IA (≥40)	Mild (40–59)	937	44.4
	Moderate (60–79)	224	10.6
	Severe (80–100)	16	0.8
Total		2,108	100.0

NIA: Non-Internet Addiction, IA: Internet Addiction.

### Prevention effect of exercise participation on internet addiction

The Chi-square test revealed that participation in leisure sports, exercise intensity, gender, and grade had a significant impact on Internet Addiction (IA) (*p* < 0.05) (Table 3). Furthermore, there were notable associations between IA and engagement in leisure sports (unadjusted RR = 0.930, 95% CI = 0.900–0.962, *p* < 0.001), exercise intensity (unadjusted RR = 0.957, 95% CI = 0.939–0.976, p < 0.001), and being female (unadjusted RR = 1.061, 95% CI = 1.033–1.091, *p* < 0.001) (Table 4).

**Table 3:** Comparison between participants with NIA and IA for category variables (*n* = 2,108).

Variables	Group	Total	NIA (n = 931)	IA (n = 1,177)	Mann Whitney test /chi square test	
					*Z* Value/*χ^2^* Value	*P* Value
Leisure Sports Participation (*n*) (%)	No	353	122 (34.6)	231 (65.4)	15.860	<0.001
	Yes	1755	809 (46.1)	946 (53.9)		
Exercise intensity (*n*) (%)	Low	1,346	552 (41.0)	794 (59.0)	20.927	<0.001
	Moderate	459	212 (46.2)	247 (53.8)		
	High	303	167 (55.1)	136 (44.9)		
Sex (*n*) (%)	Male	957	471 (49.2)	486 (50.8)	18.135	<0.001
	Female	1,151	460 (40.0)	691 (60.0)		
Double 1^st^-class Univ (*n*) (%)	Non	1875	837 (44.6)	1,038 (55.4)	1.552	0.213
	Yes	233	94 (40.3)	139 (59.7)		
University Location (*n*) (%)	East	219	97 (44.3)	122 (55.7)	2.501	0.286
	Middle	150	57 (38.0)	93 (62.0)		
	West	1739	777 (44.7)	962 (55.3)		
Discipline (*n*) (%)	Medicine	977	423 (43.3)	554 (56.7)	0.725	0.696
	STEM	598	272 (45.5)	326 (54.5)		
	Others	533	236 (44.3)	297 (55.7)		
Ethnic (*n*) (%)	Han	1973	864 (43.8)	1,109 (56.2)	1.747	0.186
	Minority	135	67 (49.6)	68 (50.4)		
Grade (*n*) (%)	1^st^	1,551	698 (45.0)	853 (55.0)	9.911	0.042
	2^nd^	297	117 (39.4)	180 (60.6)		
	3^rd^	133	49 (36.8)	84 (63.2)		
	4^th^	105	55 (52.4)	50 (47.6)		
	5^th^	22	12 (54.5)	10 (45.5)		
Age M (P_25_, P_75_)		2,108	19 (18, 20)	19 (18, 20)	−0.224	0.823

STEM: Science, Technology, Engineering and Math.

**Table 4:** Modified poisson regression.

Variables		Adjusted			Unadjusted		
		*RR* Value	*RR* 95*%* CI	*P* Value	*RR* Value	*RR* 95*%* CI	*P* Value
Leisure sports participation	No^*^						
	Yes	0.950	0.917–0.983	0.004	0.930	0.900–0.962	<0.001
Exercise intensity		0.971	0.951–0.991	0.005	0.957	0.939–0.976	<0.001
Sex	Male^*^						
	Female	1.042	1.012–1.073	0.005	1.061	1.033–1.091	<0.001
Double 1^st^-class	Non^*^						
	Yes	1.032	0.986–1.080	0.171	1.028	0.985–1.072	0.203
Univ location	East^*^						
	Middle	1.028	0.964–1.096	0.405	1.040	0.976–1.109	0.224
	West	0.997	0.951–1.045	0.904	0.998	0.954–1.043	0.913
Discipline	Medicine^*^						
	STEM	0.989	0.956–1.023	0.509	0.986	0.954–1.019	0.397
	Others	0.987	0.953–1.022	0.454	0.994	0.961–1.028	0.714
Ethnic	Han^*^						
	Minority	0.959	0.907–1.015	0.150	0.963	0.909–1.020	0.197
Grade		0.991	0.968–1.014	0.434	0.999	0.984–1.015	0.947
Age		1.003	0.988–1.018	0.705	0.998	0.989–1.008	0.765

*Referent; RR = Risk Ratio; 95% CI = 95% Confidence Interval; STEM: Science, Technology, Engineering and Math.

Moreover, a noteworthy association was found between IA and engagement in leisure sports (adjusted RR = 0.950, 95% CI = 0.917–0.983, *p* = 0.004), exercise intensity (adjusted RR = 0.971, 95% CI = 0.951–0.991, *p* = 0.005), and gender (adjusted RR = 1.042, 95% CI = 1.012–1.073, p = 0.005), even after controlling for other covariates. However, no significant associations were observed between IA and variables such as Double 1st-class University status, university location, discipline, ethnicity, grade, or age, independent of other covariates (Table 4).

## Discussion

In this prospective study, we recruited approximately 2000 undergraduate students from diverse universities located across China, including institutions from the Eastern, Middle, and Western regions. This sampling approach was carefully structured to provide a reasonable representation of the broader population of Chinese undergraduate students.

First and foremost, we noted no substantial gender disparities among the participants, signifying the absence of any selection bias. The average age of the participants, at 19 years, falls within the typical age range for undergraduate students. More specifically, among these universities, incoming freshmen typically range in age from 17 to 19 years. In relation to ethnicity, the ratio of Han Chinese participants to those from other ethnic backgrounds closely mirrored the demographic composition of the general population in China.

The universities in our study were divided into two groups: those designated as double-first class and those classified as non-double-first class institutions, based on their comprehensive assessment scores, which encompassed both teaching and research performance, including research funding. As a result, we had approximately 10% of the universities falling into the double-first class category, while the remaining 90% belonged to the non-double-first class category. This observation aligns with our finding that there were nine times as many students from non-double-first class universities as there were from double-first class universities.

While we endeavoured to recruit students evenly from various regions within the universities, we observed a higher proportion of students hailing from the Western region in comparison to the Middle and Eastern regions. This disparity can be partially attributed to the researchers’ location, as they were situated in Sichuan and Guangxi, situated in the Western or Southern part of China.

Moreover, we sought to explore potential differences between medical and non-medical students in our study, as prior research has indicated that medical students may exhibit a higher susceptibility to internet addiction (23). To mitigate selection bias, we took care to recruit a comparable number of both medical and non-medical students.

Although students from all academic years were invited to participate, it’s noteworthy that most respondents were in their first year, with the number gradually decreasing in the subsequent years. This trend can be attributed to the mounting academic demands that students encounter as they advance in their studies.

It’s worth noting that approximately 75% of students reported their participation in leisure sports activities. This notably high participation rate can be attributed to effective educational initiatives undertaken by the universities. These initiatives emphasize the crucial role of physical exercise in enhancing academic performance. The primary explanation for this improvement lies in exercise’s ability to enhance oxygen flow to the brain, leading to an increased production of brain neurotransmitters (24).

This assertion is further substantiated by other studies indicating that exercise appears to mitigate internet addiction by modulating the neurobiology of the central and autonomic nervous systems. For instance, exercise can elevate the levels of neurotrophic factors, cortisol, and neurotransmitters (18). As a result, critical brain functions such as focus, concentration, learning, memory, and stress management are notably improved (25). Our research findings are also consistent with Kim’s report, underscoring the impact of personalized individual characteristics, encompassing personal psychological and emotional factors, on internet addiction (26). However, it’s important to acknowledge that most students engaged in leisure sports activities at a moderate level, which is understandable given their academic commitments.

As anticipated, there was an inverse correlation between IA and sports activity, with this correlation linked to varying levels of sports participation. It is noteworthy that over 56% of the participating undergraduate students exhibited different levels of IA. It is reassuring to observe that most IA students fell into the moderate level category, with less than 0.8% classified as experiencing severe IA. From a statistical perspective, the data derived from the Chi-square test confirmed the presence of an inverse correlation between participation in leisure sports and IA, consistent with its connection to the intensity of sports involvement.

We noted that women exhibited a higher susceptibility to IA in comparison to men, which could be attributed to their relatively lower levels of physical exercise. This observation aligns with the earlier finding of an inverse correlation between sports participation and IA. When considering academic performance and university status, we found no significant difference in IA prevalence between students from double-first-class and non-double-first-class universities. This suggests that IA is not directly associated with academic performance or the status of the universities.

Furthermore, we discovered no correlation between geographic location and the occurrence of IA. While there might be variations in internet coverage between regions, such as rural mountainous areas compared to metropolitan areas, it’s important to note that internet access is nearly universally comprehensive across all universities. Consequently, the availability of internet access does not seem to be a significant contributing factor to the development of IA among students across different geographic locations.

No significant difference in IA was observed between medical and non-medical students, implying that IA is not linked to the specific academic discipline’s students pursue. This finding suggests that, despite potential variations in academic-related stressors, the campus environments appear to be similar and do not substantially contribute to the development of IA. Furthermore, our study revealed no significant disparity in IA among students from different ethnic backgrounds. This can be attributed to the modernization of societies and the widespread accessibility of modern technologies. In China, almost every student can access the internet at a relatively low cost, which diminishes the influence of ethnicity on IA. This is supported by the report demonstrating that Chinese education is gradually shifting towards online teaching among college students, making internet access more affordable (27).

It’s also noted that freshmen students were more prone to experiencing IA. This tendency may be attributed to the culmination of their long-term preparations for university entrance, combined with the stress arising from family expectations. Upon entering the university, they often find themselves in a more independent and unsupervised environment. However, as students’ progress through their academic journey, they gradually mature and assume more responsibilities, which tends to result in a decline in IA. This observation aligns with the previously mentioned inverse correlation between age and IA, especially during adolescence (28), some of which may be associated with structural changes in the brain (29).

While the data collected was from March 2023, it is important to note that the students’ performance occurred during the COVID-19 lockdown period, spanning from 2020 to 2022. We did not compare the outcomes before and after the COVID-19 lockdown, which is a consideration for future research. In summary, our data suggests that physical exercise, gender, and academic year have a significant impact on IA.

## Data availability statement

The raw data supporting the conclusions of this article will be made available by the authors, without undue reservation.

## Ethics statement

The studies involving humans were approved by The Second Hospital, Guilin Medical University. The studies were conducted in accordance with the local legislation and institutional requirements. Written informed consent for participation in this study was provided by the participants' legal guardians/next of kin.

## Author contributions

PC: Data curation, Funding acquisition, Methodology, Writing – original draft. JW: Data curation, Methodology, Writing – review & editing. PY: Data curation, Methodology, Writing – review & editing. XF: Methodology, Investigation, Writing – review & editing. GY: Methodology, Data curation, Writing – original draft. CH: Methodology, Data curation, Writing – review & editing. XC: Data curation, Investigation, Writing – original draft. BH: Writing – review & editing. SB: Writing – review & editing, Supervision. SL: Conceptualization, Writing – review & editing, Funding acquisition, Project administration.

## Footnotes

^1^ https://www.wjx.cn/
